# Supplementary figures and images for: Extended spectrum β-lactamase (ESβL)-producing E. coli causing urinary tract infection among pregnant women and pediatric patients in public hospitals in northern Jordan
Source: PLoS One. 2025 Mar 31;20(3):e0320292. doi: 10.1371/journal.pone.0320292 (PMC11957286; doi:10.1371/journal.pone.0320292)

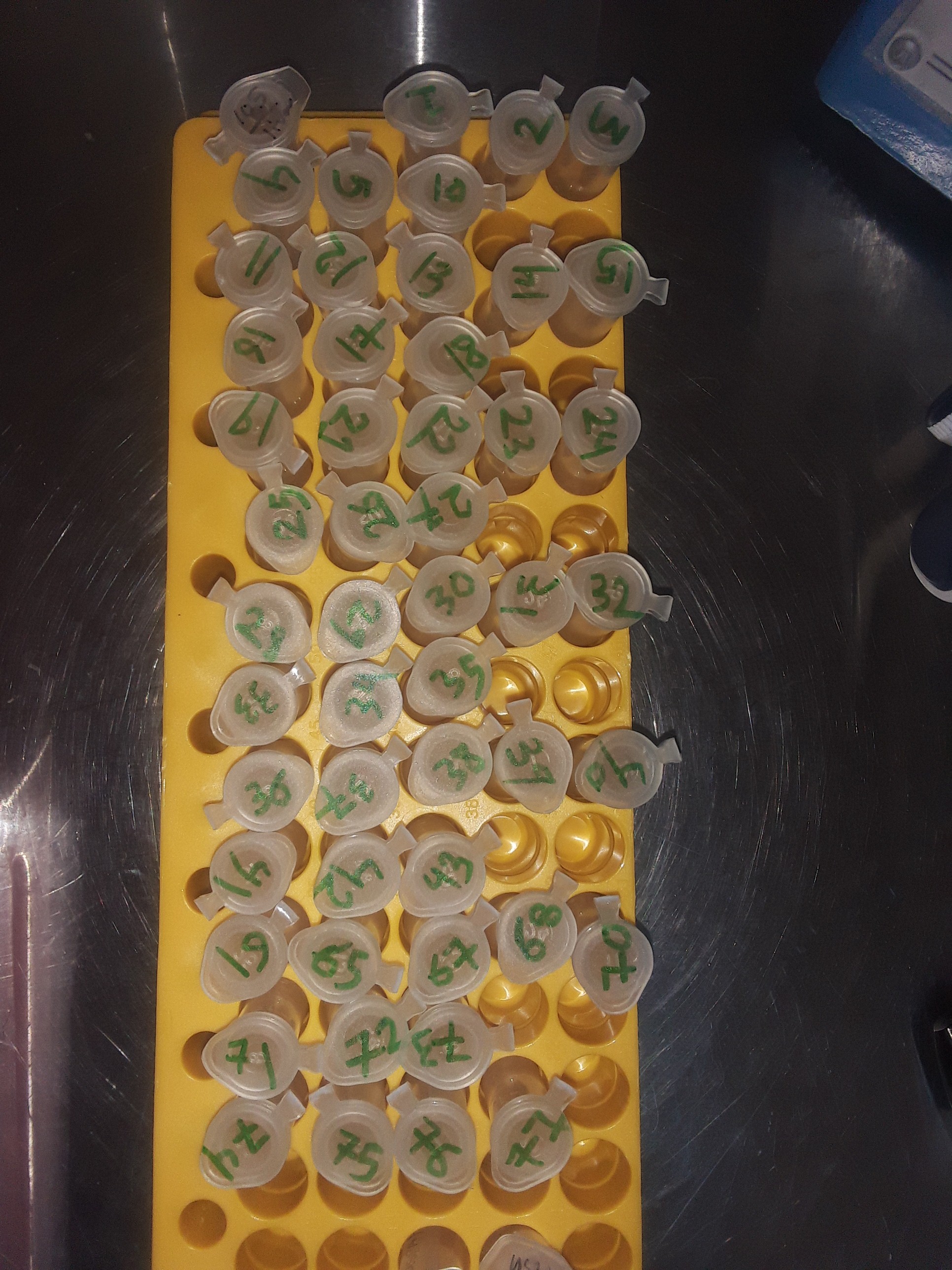

Supplement: S1 Data — (ZIP) [file pone.0320292.s001.zip › 20211229_102532.jpg]

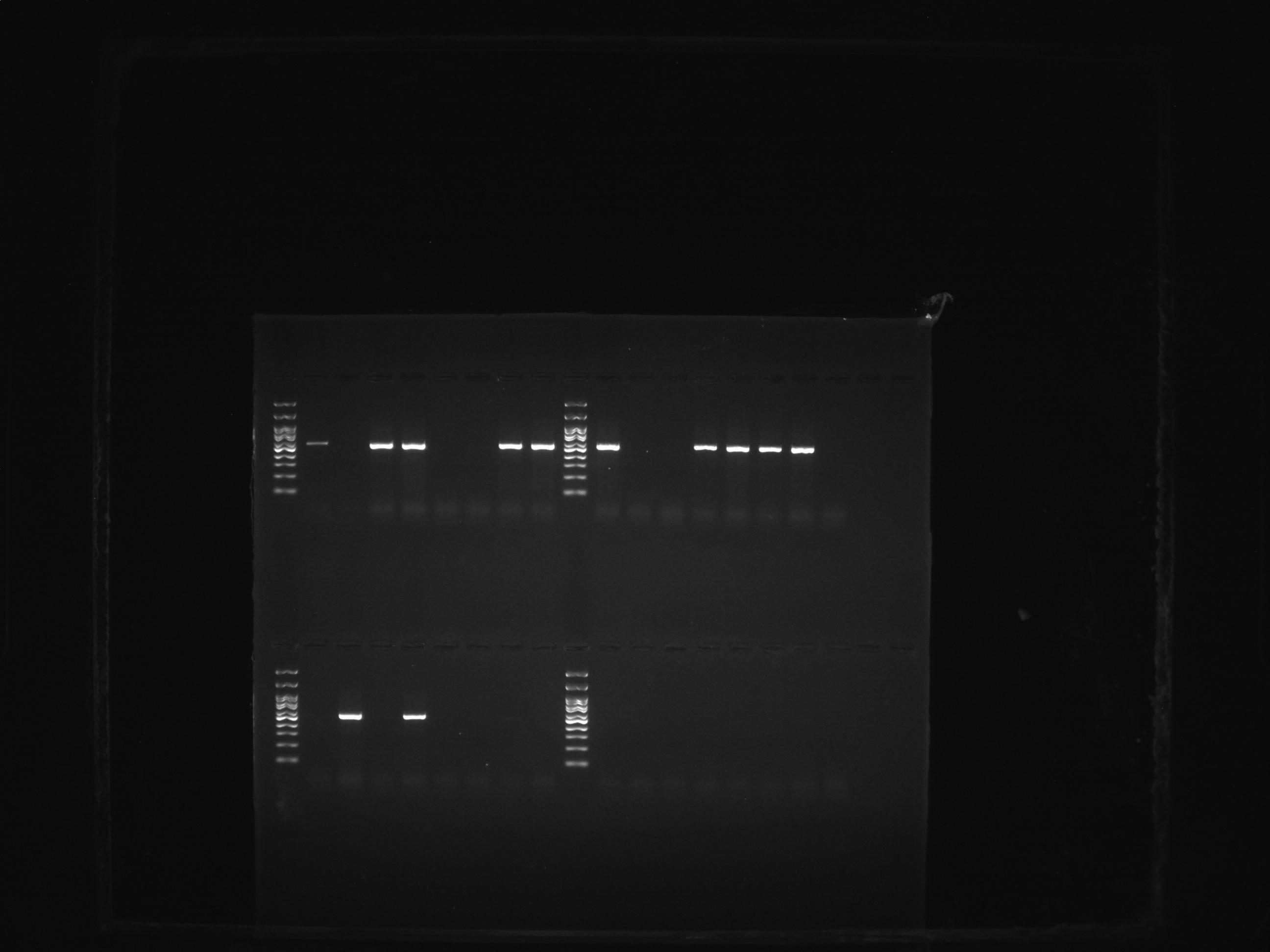

Supplement: S1 Data — (ZIP) [file pone.0320292.s001.zip › CTXM/ctxm s1 to s4.jpg]

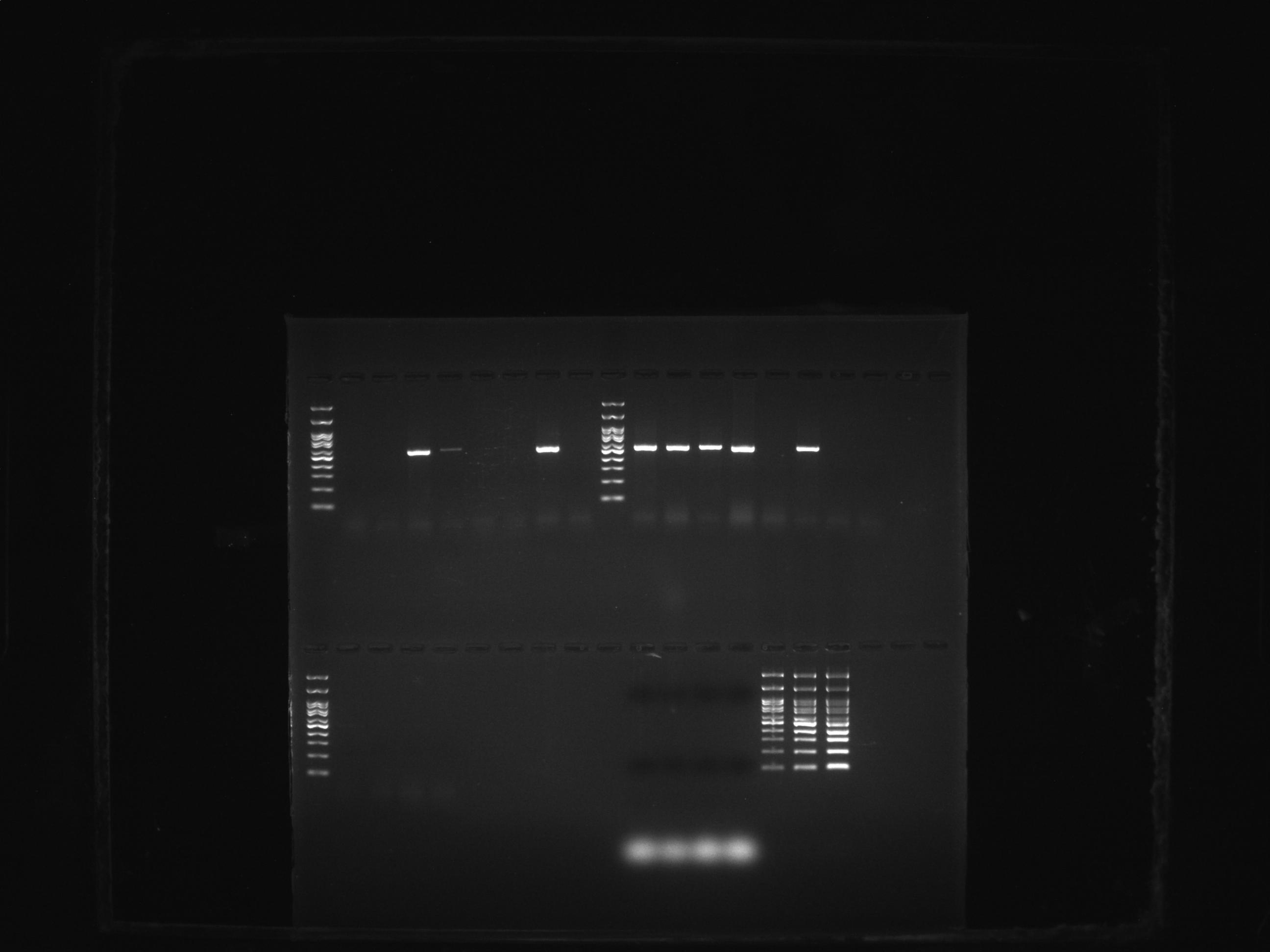

Supplement: S1 Data — (ZIP) [file pone.0320292.s001.zip › CTXM/ctxm s5 to s7.jpg]

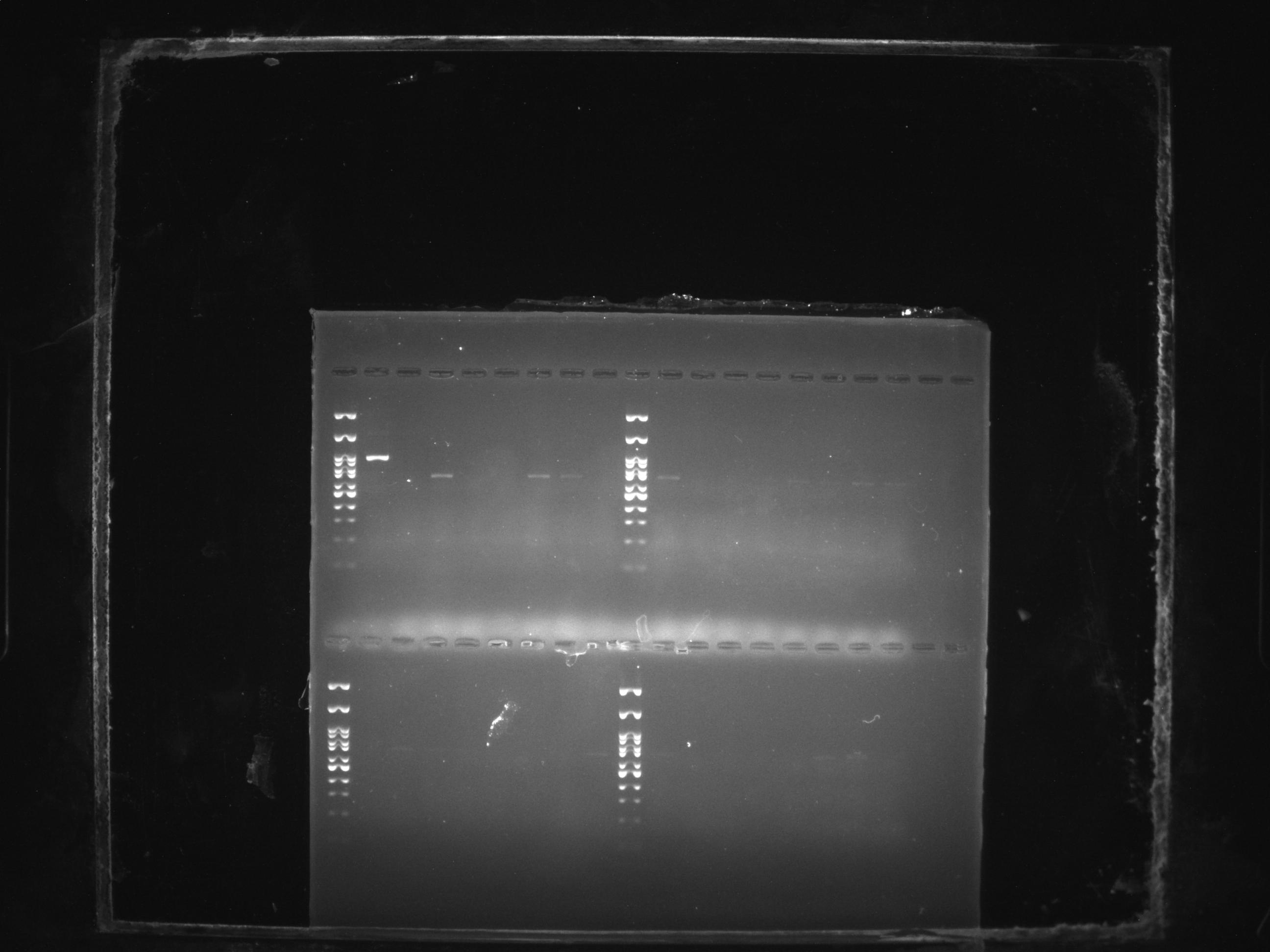

Supplement: S1 Data — (ZIP) [file pone.0320292.s001.zip › SHV/shv r1 to r4.jpg]

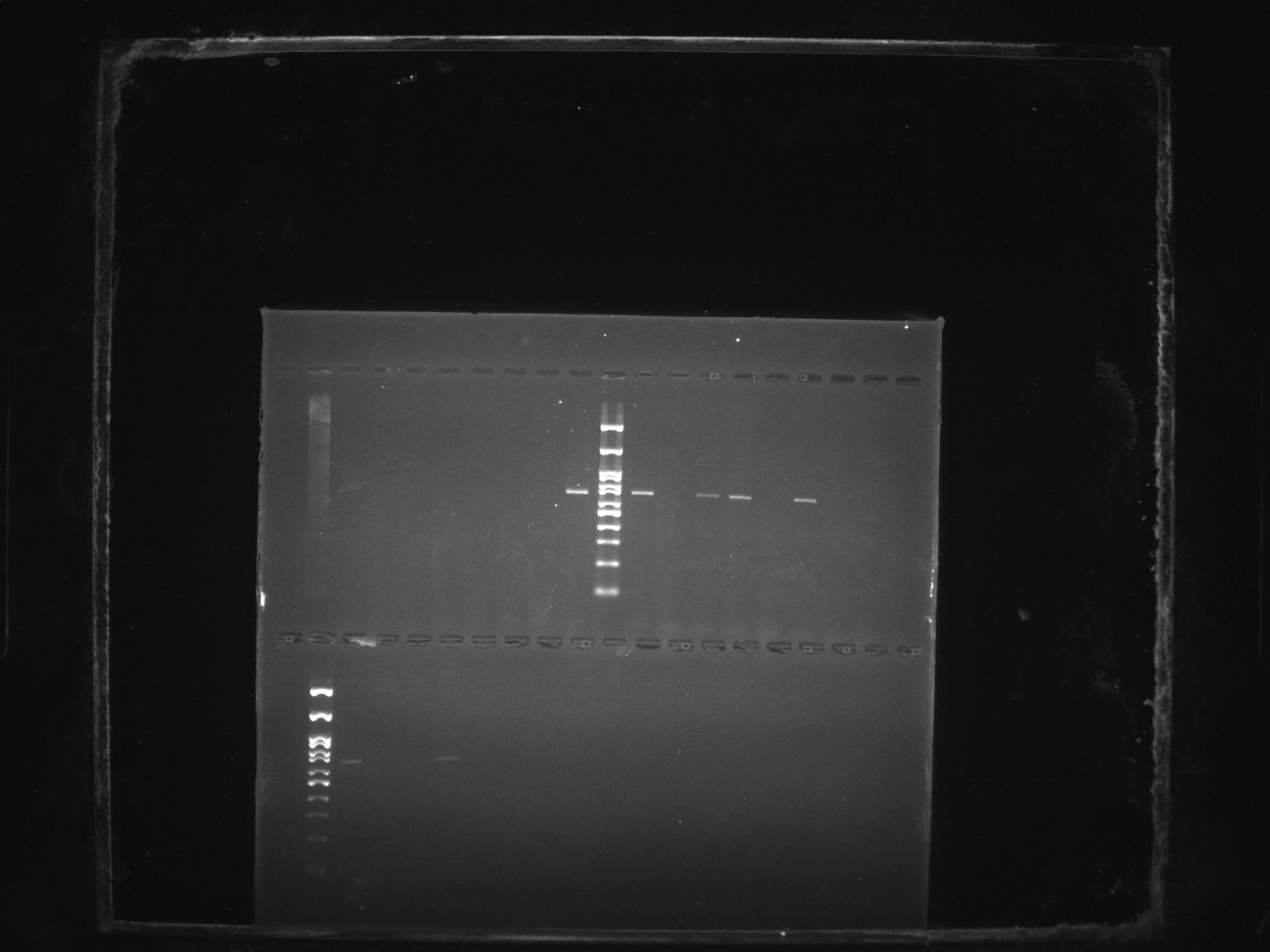

Supplement: S1 Data — (ZIP) [file pone.0320292.s001.zip › SHV/shv r5 to r7.jpg]

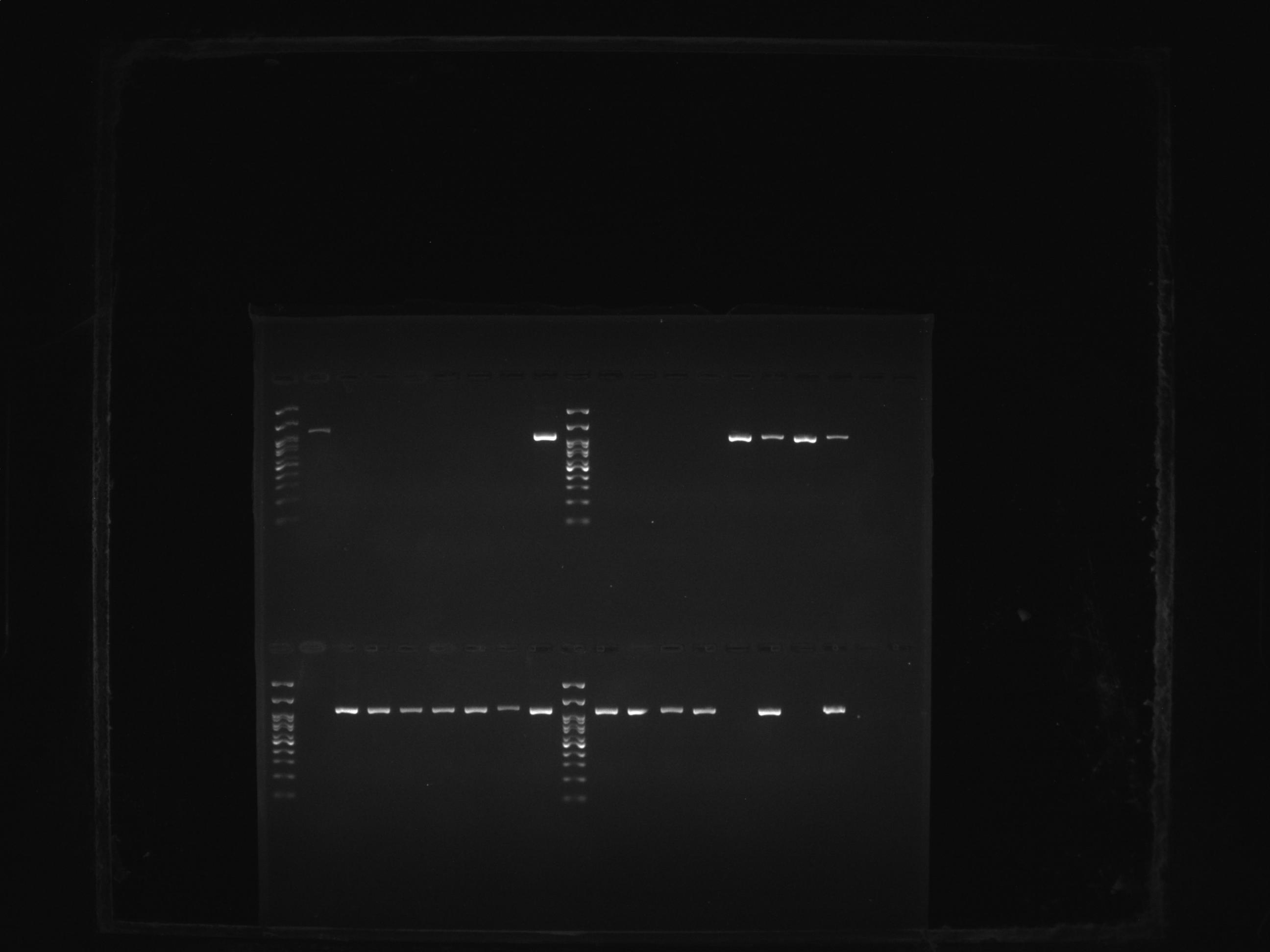

Supplement: S1 Data — (ZIP) [file pone.0320292.s001.zip › TEM/tem r1 to r4.jpg]

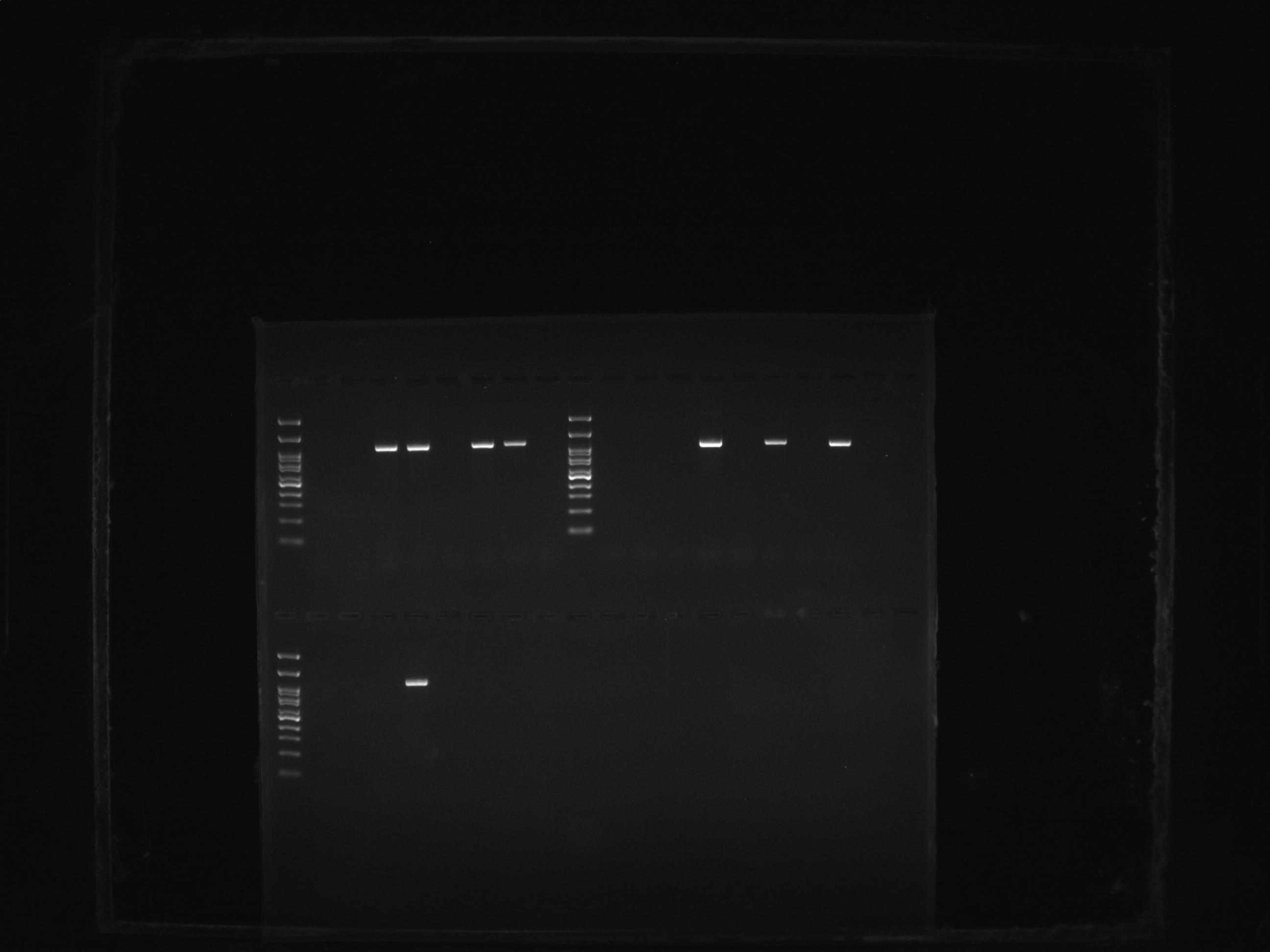

Supplement: S1 Data — (ZIP) [file pone.0320292.s001.zip › TEM/tem r5 to r7.jpg]

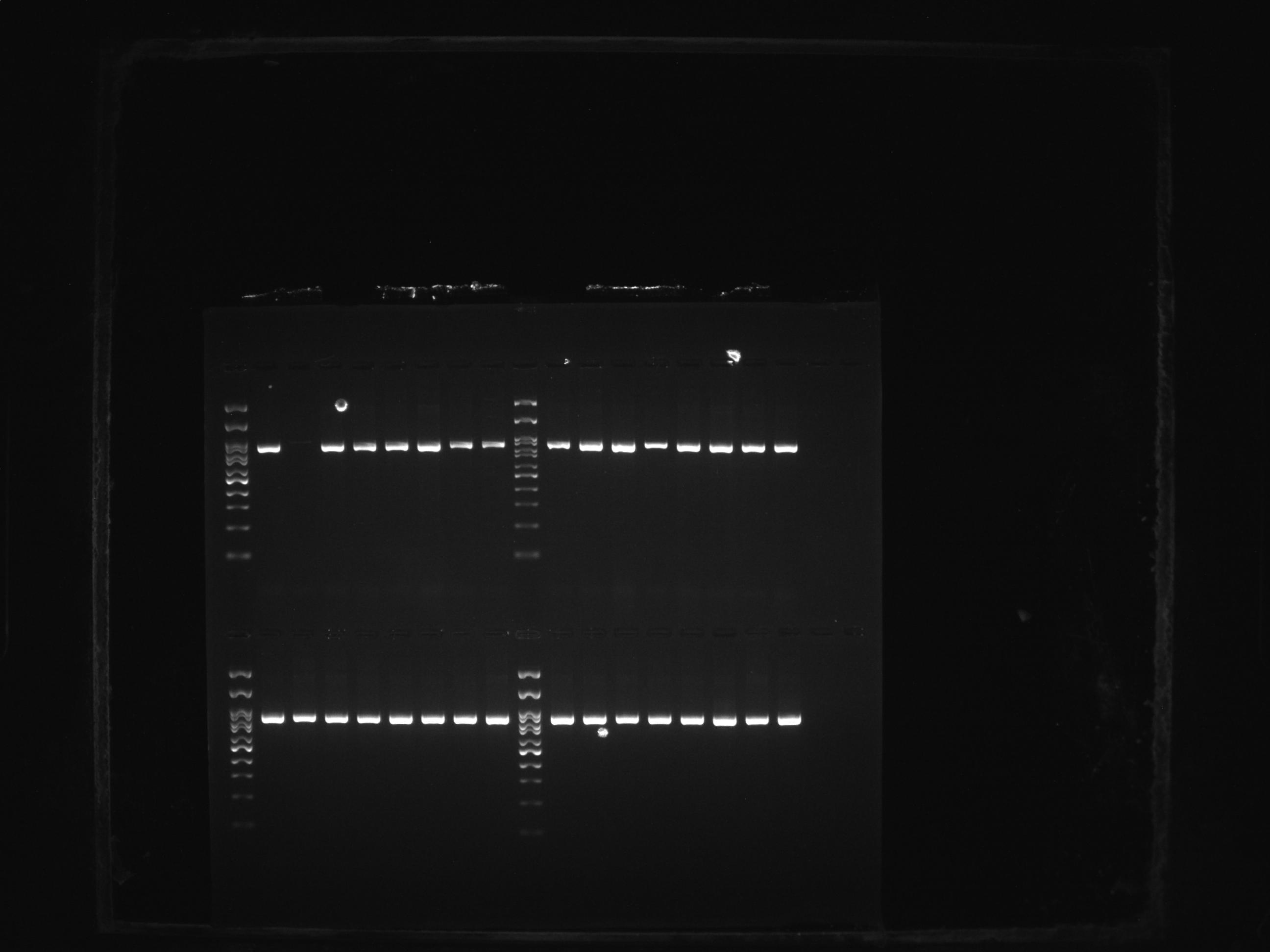

Supplement: S1 Data — (ZIP) [file pone.0320292.s001.zip › USPA/uspA S1 To S4.jpg]

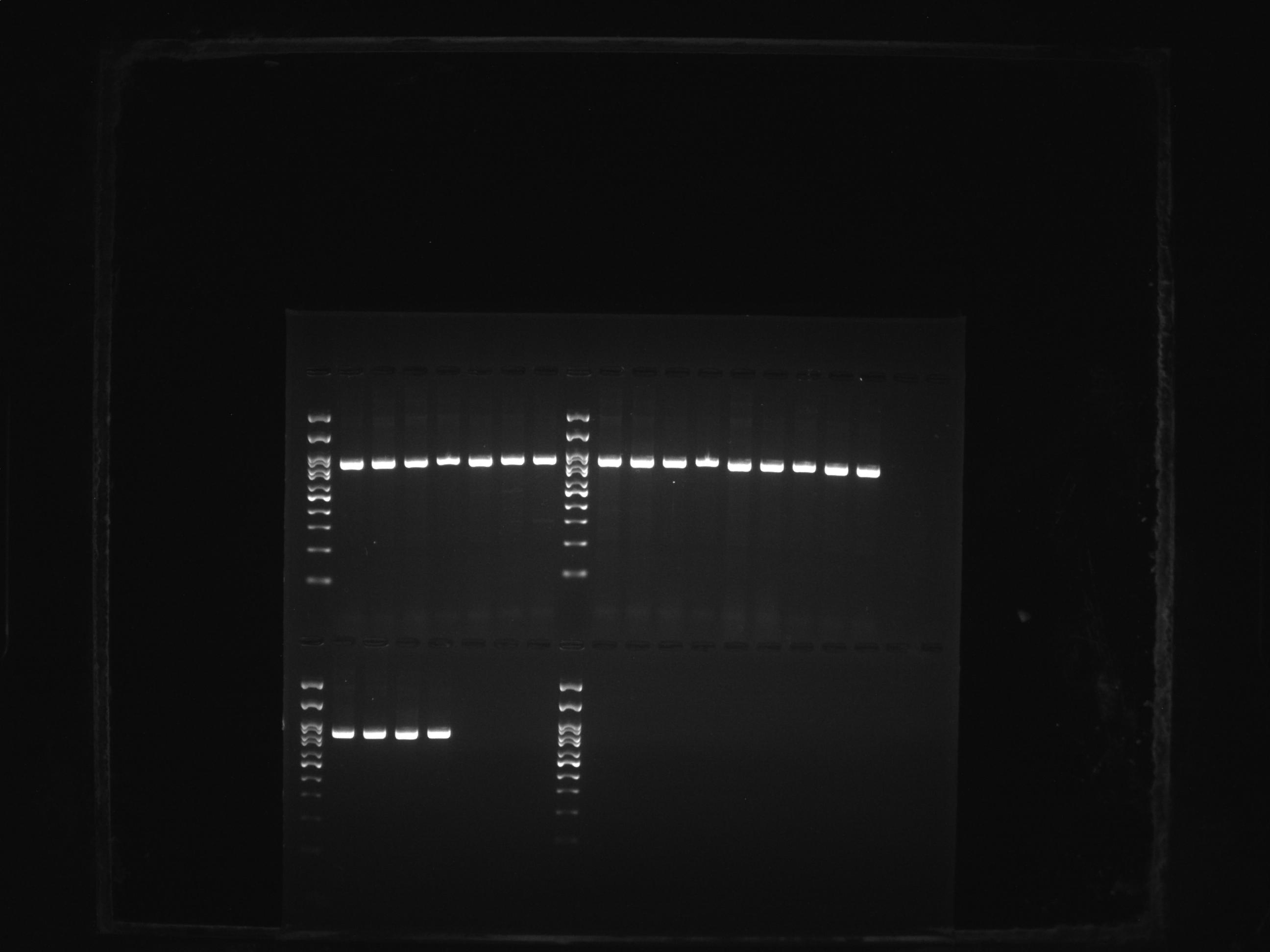

Supplement: S1 Data — (ZIP) [file pone.0320292.s001.zip › USPA/uspA S5 To S7.jpg]
