# Supplementary material for: Extended spectrum β-lactamase (ESβL)-producing E. coli causing urinary tract infection among pregnant women and pediatric patients in public hospitals in northern Jordan
Source: PLoS One. 2025 Mar 31;20(3):e0320292. doi: 10.1371/journal.pone.0320292 (PMC11957286; doi:10.1371/journal.pone.0320292)
Supplement: Attachment — Submitted filename: 2nd Review report for Plose one.pdf [file pone.0320292.s008.pdf]

Thank you for your response and for revising your manuscript based on my previous comments. However, some critical issues have not been fully addressed in the revised manuscript. Please make the necessary modifications based on the following comments:

Abstract

Background: The background lacks sufficient detail. The sentence "Based on clinical presentation and diagnosis, UTI is classified into upper and lower UTI" should be removed. Focus instead on the identified gaps in existing research or clinical practice that led to the study's title.

Introduction: The introduction needs more clarity regarding the specific gaps that led to the selection of this study title. Clearly state the issues or knowledge gaps that prompted the research question and title.

Methods:

- Sample Size Determination: The rationale for selecting 70 isolates is unclear. Please explain why 70 patients (isolates) were chosen and whether any statistical methods were used for sample size calculation.
- Data Entry and Analysis: Specify the software used for data entry and analysis (e.g., EpiData, SPSS, Stata). Provide details of the analysis methods to ensure the results are valid.
- Inclusion and Exclusion Criteria: The criteria for participant inclusion and exclusion need clarification. Specify the symptoms required for inclusion and whether bacterial growth was from study participants or existing data.
- Patient Population: Clarify the source of the 70 isolates (e.g., hospital patients, outpatient clinics) and describe the population from which they were collected.
