# Supplementary material for: Extended spectrum β-lactamase (ESβL)-producing E. coli causing urinary tract infection among pregnant women and pediatric patients in public hospitals in northern Jordan
Source: PLoS One. 2025 Mar 31;20(3):e0320292. doi: 10.1371/journal.pone.0320292 (PMC11957286; doi:10.1371/journal.pone.0320292)
Supplement: Attachment — Submitted filename: response to editors and reviewers comments 9.12.24.docx [file pone.0320292.s007.docx]

Additional Editor Comments:

The editor's comments, and the reviewers’ comments are highly appreciated, and valued. Please find the response point by point and the changes made. We did our best effort to adjust and improve the quality of this article. Your insight and input is much appreciated.

- The manuscript was significantly improved, but it requires a through revision. Done with care.
- In the abstract, use at least the two versions at the same time for abbreviations, e.g, Urinary tract infections (UTI),……. Done.
- There was no ‘objective’ of the study in the abstract part. Added
- When, where and from whom the study was conducted? It should be included in both the abstract and the methods part. Added
- How could you collect the sample? What type of urine sample was collected? Added
- In the abstract and result, the absolute number (numerator and denominator) is needed together with the percentage. For example, in the abstract for *E. coli* A/B (71.4%). Done.
- Your conclusion is explanation. Please make it conclusion, and try to decrease the numbers. Done.
- All key words are abbreviations, please write their long form	Done.
- Paragraphing was needed throughout the document, especially in the introduction and discussion part. Done.
- It is better you organize the ‘Materials and Methods’ part as follows Done.

Materials and Methods

Study area and setting

Study design and period

Study population

Sample size determination and sampling technique (if any)

Samples collection and bacterial isolation


Ethical Considerations

- A separate ‘Ethical consideration’ statement is needed. Will upload separately.
- Table 1 was not needed. You can write it as a statement. The antimicrobials used were ampicillin (AMP, 10µg), norfloxacin (NOR, 10µg), …………….. Removed. Table 1.
- Line 123: **Bacterial isolates,** not bacterial isolation and identification Done.
- Line 124: A sentence cannot start by number and abbreviation. ‘70 bacterial isolates were identified…..’ It is better written as, ‘Seventy bacterial isolates were identified…..’ Done.
- Line 150: The following should be below the tables. R: Resistant; S: Susceptible; I: Intermediate. Changed The title to resistance percentages.
- Line 160: Table 6: MAR values for all tested isolates. Please write the long form of “MAR” and the description should be full in terms of person, place and time. Done.
- Line 163: ……was the most frequent (n= 26, 52%) followed by ESβLCTXM (N= 19, 38%) (Figure 1 & 2). Done.
- Critically revise citations of figures and tables. Done.
- Funding, Availability of data and materials, Competing interests and Acknowledgments should be written in standard English. Done.
- Tables are too poor and you have to prepare them properly. Hopefully. Done. And adjusted.
- The references were not written properly. All references should be written correctly according to Vancouver style. Hopefully. Done. And adjusted.
- Use the alignment ‘Justify’ for the text throughout the manuscript Done.
- The reference writing was not correct and need revision Hopefully. Done. And adjusted.
- Follow the standard binomial nomenclature, italize journal name and the word ‘et al’ done
- Follow the guideline for manuscript writing protocol for PLoS One. Hopefully. Done. And adjusted.

[Note: HTML markup is below. Please do not edit.]

Reviewers' comments:

Reviewer's Responses to Questions

**Comments to the Author**

1. If the authors have adequately addressed your comments raised in a previous round of review and you feel that this manuscript is now acceptable for publication, you may indicate that here to bypass the “Comments to the Author” section, enter your conflict of interest statement in the “Confidential to Editor” section, and submit your "Accept" recommendation.

Reviewer #1: (No Response)

2. Is the manuscript technically sound, and do the data support the conclusions?

The manuscript must describe a technically sound piece of scientific research with dataooypdd that supports the conclusions. Experiments must have been conducted rigorously, with appropriate controls, replication, and sample sizes. The conclusions must be drawn appropriately based on the data presented.

Reviewer #1: Partly

3. Has the statistical analysis been performed appropriately and rigorously?

Reviewer #1: Yes

4. Have the authors made all data underlying the findings in their manuscript fully available?

The [PLOS Data policy](http://track.editorialmanager.com/CL0/http:%2F%2Fwww.plosone.org%2Fstatic%2Fpolicies.action%23sharing/1/010f01936df04a30-e8b08d3d-cab2-4bfc-a037-eab2dca42f0c-000000/bvnP3jVOF4WdR2DLoGi6Kn8XhwUgqtbNkPuJMLzUvT0=186) requires authors to make all data underlying the findings described in their manuscript fully available without restriction, with rare exception (please refer to the Data Availability Statement in the manuscript PDF file). The data should be provided as part of the manuscript or its supporting information, or deposited to a public repository. For example, in addition to summary statistics, the data points behind means, medians and variance measures should be available. If there are restrictions on publicly sharing data—e.g. participant privacy or use of data from a third party—those must be specified.

Reviewer #1: Yes

5. Is the manuscript presented in an intelligible fashion and written in standard English?

PLOS ONE does not copyedit accepted manuscripts, so the language in submitted articles must be clear, correct, and unambiguous. Any typographical or grammatical errors should be corrected at revision, so please note any specific errors here.

Reviewer #1: No

6. Review Comments to the Author

Please use the space provided to explain your answers to the questions above. You may also include additional comments for the author, including concerns about dual publication, research ethics, or publication ethics. (Please upload your review as an attachment if it exceeds 20,000 characters)

- Reviewer #1: Reviewer comment
  The authors have addressed most of the previous reviewer comments; however, the manuscript still has many problems.
  Line 1: insert symbol for beta before word lactamase Done.
  Line 23-24: remove this sentence “and is responsible for 60-80% of these infections.” Done.
  Line 24-25: replace this sentence “UTI is classified based on clinical presentation Done.
- and diagnosis into upper UTI and lower UTI” with this: “based on the clinical presentation and diagnosis UTI classified into upper and lower UTI” Done.
  Line 32,33,39 ,126,127,133 and other parts of the manuscript write the scientific name of bacterial isolates correctly: species name is not started in capital letter: write Klebsiella pneumoniae correctly Done.
  "Line 40 states, 'Multiple antibiotic resistance.' What does it mean? Does it refer to 'multidrug resistant'?" Done.
  Line 41: “0.62” change it in to percentage Done.
  Line 37-44: The conclusion section in the abstract appears to present specific Done.

results rather than a conclusion. Please provide a general conclusion of the study based on your results. Done.

I started assessing the manuscript line by line, but I couldn’t reach the end because it is full of errors
General comments
Some scientific name of bacterial isolates note written correctly. Done.
There no definition or criteria for multidrug resistance isolates Done.
Conclusion statement in the abstract and the conclusion section are completely different. Done.
In the method section
The ethical statement is included in the study design section Done.
Please list the names of the antibiotics used in a statement rather than in a table. Done.
There no reference putted for the for CLSI in the method section
Please provide the detailed PCR conditions in a statement rather than in a table, starting from the initial denaturation to the final extension.
The author comparing the prevalence of bacterial isolates and antibiotic resistance results among different groups without including statistical significance values
In some cases, the author uses only the genus, while in others, species of isolates are used. See Tables 4, 5, 6, and others. Done.

Finally, although this study used advanced methods like VITAKE and PCR, the authors need a well-experienced microbiologist to understand the results and rewrite the manuscript. Moreover, the author needs individuals for proofreading in English for grammar, spelling, coherence, and flow of sentence. Hopefully done and improved

7. PLOS authors have the option to publish the peer review history of their article ([what does this mean?](https://track.editorialmanager.com/CL0/https:%2F%2Fjournals.plos.org%2Fplosone%2Fs%2Feditorial-and-peer-review-process%23loc-peer-review-history/1/010f01936df04a30-e8b08d3d-cab2-4bfc-a037-eab2dca42f0c-000000/ti96FWQFYt2x_E3yQvQHhwMVcHk8TcdTlv2kI_XEDR0=186)). If published, this will include your full peer review and any attached files.


If you choose “no”, your identity will remain anonymous but your review may still be made public.


**Do you want your identity to be public for this peer review?** For information about this choice, including consent withdrawal, please see our [Privacy Policy](https://track.editorialmanager.com/CL0/https:%2F%2Fwww.plos.org%2Fprivacy-policy/1/010f01936df04a30-e8b08d3d-cab2-4bfc-a037-eab2dca42f0c-000000/PICQ0yW7u3p1DSoGBc0BiTm1WqHZJIKE5kRCvQW9BB0=186).

Reviewer #1: No

[NOTE: If reviewer comments were submitted as an attachment file, they will be attached to this email and accessible via the submission site. Please log into your account, locate the manuscript record, and check for the action link "View Attachments". If this link does not appear, there are no attachment files.]

While revising your submission, please upload your figure files to the Preflight Analysis and Conversion Engine (PACE) digital diagnostic tool, [https://pacev2.apexcovantage.com/](https://track.editorialmanager.com/CL0/https:%2F%2Fpacev2.apexcovantage.com%2F/1/010f01936df04a30-e8b08d3d-cab2-4bfc-a037-eab2dca42f0c-000000/VReHK92sf9_DdQ5jrPpMlkj0hl89jje3-HMy_UldKNE=186). PACE helps ensure that figures meet PLOS requirements. To use PACE, you must first register as a user. Registration is free. Then, login and navigate to the UPLOAD tab, where you will find detailed instructions on how to use the tool. If you encounter any issues or have any questions when using PACE, please email PLOS at [figures@plos.org](mailto:figures@plos.org). Please note that Supporting Information files do not need this step.
