# Supplementary material for: Extended spectrum β-lactamase (ESβL)-producing E. coli causing urinary tract infection among pregnant women and pediatric patients in public hospitals in northern Jordan
Source: PLoS One. 2025 Mar 31;20(3):e0320292. doi: 10.1371/journal.pone.0320292 (PMC11957286; doi:10.1371/journal.pone.0320292)
Supplement: Attachment — Submitted filename: Response to reviewrs Plos One UTI Ecoli.docx [file pone.0320292.s006.docx]

Dear Tebelay Dilnessa PLOS ONE Editor

Thank you for the chance to edit our submission and re submit with the changes you and honourable reviewers suggested.

Please find response to the editors and reviews comments point by point.

1. Journal requirements

Up to our best knowledge, the manuscripts was made to meet PLOS ONE style requirements, including those for file naming and we tried to follow PLOS ONE style templates as much as we could and we adjusted any overlapping texts and cited any information that we added into our article. Up to our best knowledge, we included captions for the figures at the end of our manuscript, and we cited that in the texts when it was applicable.

To our best knowledge, the paper or the manuscripts writing has been improved to the scientific standard, and we tried to follow The PLOS ONE writing protocol. The laboratory method has been edited and written in a better way and the tables and figures description has been edited and explained in a better way. The citations, where edited and corrected to the best possible way and follow the protocol of PLOS ONE. Conclusions and recommendations were added and figures have been prepared and uploaded as a separate files. Funding and availability of data, conflict of interest and acknowledgement has been added to the manuscript.

Response to reviewers

Reviewer 1 comments. The manuscript and the results section have been edited and the methodology has been explained thoroughly and the sample size, date and place of the research has been added to the methodology section.

Statistical analysis has been reviewed and more information added to make it clearer and more understandable. All the Data is included in the manuscript and specific details and data will be available and provided upon request if needed. The manuscript has been edited and written in a better scientific way and has been reviewed multiple times. Grammar and typos has been checked and corrected up to our best knowledge.

Response to reviewer one comments the methodology section has been edited ,study area, study design and the period of the study has been added and inclusion and exclusion criteria as well. Reference guideline for antibiotic resistance and PCR conditions and data analysis has been included in the methodology section.

The results section has been edited to make it more clear and more understandable. The tables have been edited and more information added such as the number and the frequency, the tables and the figures legends have been edited and a conclusion section has been added as well.

In response to reviewer 2 comments, the results section has been expanded and explained in a better way. All the suggested corrections where corrected line by line. The bacterial identification criteria is added and CLSO guidelines has been included. A conclusion section has been added and the figures have been adjusted and added as a separate file.

Dear Editor

We have worked hard to improve our manuscript and adjusted all suggested comments to bring this manuscript up to the standard of your reputable journal, please kindly review our revised manuscript and we do hope that it will be accepted in your reputable journal.

Kind regards
