# Supplementary material for: Extended spectrum β-lactamase (ESβL)-producing E. coli causing urinary tract infection among pregnant women and pediatric patients in public hospitals in northern Jordan
Source: PLoS One. 2025 Mar 31;20(3):e0320292. doi: 10.1371/journal.pone.0320292 (PMC11957286; doi:10.1371/journal.pone.0320292)
Supplement: Attachment — Submitted filename: Response to reviewers 11.2.25.docx [file pone.0320292.s010.docx]

Dear Editor;

The editor's comments and the reviewers’ comments are highly appreciated and valued. Please find the response point by point and the changes made. We did our best effort to adjust and improve the quality of this article. Your insight and input is much appreciated.

Editors’ comments

Journal Requirements:

Please review your reference list to ensure that it is complete and correct. If you have cited papers that have been retracted, please include the rationale for doing so in the manuscript text, or remove these references and replace them with relevant current references. Any changes to the reference list should be mentioned in the rebuttal letter that accompanies your revised manuscript. If you need to cite a retracted article, indicate the article’s retracted status in the References list and also include a citation and full reference for the retraction notice. Dear editor, I have gone through the references one by one and updated the reference list, I did not find any retracted articles, I have updated the CDC and WHO references to more recent ones and I have noticed the list was shifted in the old manuscript version by 1-2 numbers by mistake, I do apologize for that mistake, it was rectified and double checked. All the references are correct and numbered correctly.

Additional Editor Comments:

- Line 27: Methods: This study was conducted in Public-Teaching Hospitals in…… corrected
- Why you use outdated CLSI guidelines (ref. 32 and 33) than updated CLSI, 2023 and 2024 guidelines. Updated as advised
- These must be changed by updated one. ‘32. CLSI. Performance Standards for Antimicrobial Susceptibility Testing. 27th ed. CLSI supplement M100. Wayne, PA: Clinical and Laboratory Standards Institute; 2017; 33. CLSI. Performance Standards for Antimicrobial Susceptibility Testing. 23th ed. CLSI supplement M100. Wayne, PA: Clinical and Laboratory Standards Institute; 2013’. Done as advised much appreciated
- Tables and figures descriptions should be well explanatory in terms of person, place and time. Went through them again and hopefully changed to make it more clear.
- Figure 2 should be prepared without title/figure description (It said Figure 1: Detection of ESBL in *E. coli* isolates. Even the figure is number 2, but you said Figure 1. Corrected, thank you
- You did not give response to the editor. I repeatedly told you that you have to follow the journal manuscript writing protocol. Dear Editor. I went through the Journal publication guidelines Section by section. I've tried my best to make it compatible with the journal publication policies. Including headings, subheadings, sectioning and referencing. Hopefully, I made all the changes that is requested by the editor.

Reviewers comments,

Thank you for your response and for revising your manuscript based on my previous comments.

However, some critical issues have not been fully addressed in the revised manuscript. Please

make the necessary modifications based on the following comments:

**Abstract**

**Background:** The background lacks sufficient detail. The sentence "Based on clinical

presentation and diagnosis, UTI is classified into upper and lower UTI" should be removed.

Focus instead on the identified gaps in existing research or clinical practice that led to the study’s

title. Dear reviewer, the sentence specified has been removed. And the structure of the abstract. Has been modified to clarify the knowledge gaps.

**Introduction:** The introduction needs more clarity regarding the specific gaps that led to the

selection of this study title. Clearly state the issues or knowledge gaps that prompted the research

question and title. The introduction has been modified and a sentences has been added to make it more clear and to identify specific gaps that led to the selection of this study highlighted in the revised manuscript.

**Methods:**

• Sample Size Determination: The rationale for selecting 70 isolates is unclear. Please

explain why 70 patients (isolates) were chosen and whether any statistical methods were

used for sample size calculation. The sample size determination was determined by the number of samples we could collect in the duration of the study that fits the criteria of inclusion. All patients (pregnant women and children) who met the criteria of getting UTI’s (had symptoms and culture resulted in a bacterial growth) were included in the study, seventy UTI cases were covered in this study. This was added to the methods section

• Data Entry and Analysis: Specify the software used for data entry and analysis (e.g.,

EpiData, SPSS, Stata). Provide details of the analysis methods to ensure the results are

valid.The study was carried out during the period from the 1^st^ of March 2024 to the 5^th^ of April 2024 and all analysis was done on Microsoft Excel.

• Inclusion and Exclusion Criteria: The criteria for participant inclusion and exclusion need

clarification. Specify the symptoms required for inclusion and whether bacterial growth

was from study participants or existing data. The sample size determination was determined by the number of samples we could collect in the duration of the study that fits the criteria of inclusion. All patients (pregnant women and children) who met the criteria of getting UTI’s (had symptoms and culture resulted in a bacterial growth) were included in the study, seventy UTI cases were covered in this study. This was added to the methods section

• Patient Population: Clarify the source of the 70 isolates (e.g., hospital patients, outpatient

clinics) and describe the population from which they were collected. A hospital-based prospective study was carried out in public-teaching Hospitals in northern Jordan with a total capacity of 400 beds (Badieah and Rahma Hospitals). 70 urine samples were collected from pediatrics and female outpatients. The study was carried out during the period from the 1^st^ of March 2024 to the 5^th^ of April 2024 and all analysis was done on Microsoft Excel. This aws added to the methods section

| Comment | Response |
| --- | --- |
| The background lacks sufficient detail. The sentence "Based on clinical  presentation and diagnosis, UTI is classified into upper and lower UTI" should be removed. Focus instead on the identified gaps in existing research or clinical practice that led to the study’s  title | Done, Removed and More info were added |
| The introduction needs more clarity regarding the specific gaps that led to the  selection of this study title. Clearly state the issues or knowledge gaps that prompted the research  question and title. | Added Highlighted in Green |
| The rationale for selecting 70 isolates is unclear. Please  explain why 70 patients (isolates) were chosen and whether any statistical methods were  used for sample size calculation. | A hospital-based prospective study was carried out in public-teaching Hospitals in northern Jordan with a total capacity of 400 beds (Badieah and Rahma Hospitals). 70 urine samples were collected from pediatrics and female outpatients. The study was carried out during the period from the 1^st^ of March 2024 to the 5^th^ of April 2024 and all analysis was done on Microsoft Excel. |
| Specify the software used for data entry and analysis (e.g.,  EpiData, SPSS, Stata). Provide details of the analysis methods to ensure the results are  valid. | Added Highlighted in Green  “and all analysis was done on Microsoft Excel” |
| The criteria for participant inclusion and exclusion need  clarification. Specify the symptoms required for inclusion and whether bacterial growth  was from study participants or existing data. | The sample size determination was determined by the number of samples we could collect in the duration of the study that fits the criteria of inclusion. All patients (pregnant women and children) who met the criteria of getting UTI’s (had symptoms and culture resulted in a bacterial growth) were included in the study, seventy UTI cases were covered in this study. This was added to the methods section |
| clarify the source of the 70 isolates (e.g., hospital patients, outpatient  clinics) and describe the population from which they were collected. | Added Highlighted in Green |
| Line 27: Methods: This study was conducted in Public-Teaching Hospitals in…… | Changed |
| Why you use outdated CLSI guidelines (ref. 32 and 33) than updated CLSI | Updated to CLSI 2024 |
| Tables and figures descriptions should be well explanatory in terms of person, place and time.  Figure 2 should be prepared without title/figure description (It said Figure 1: Detection of ESBL in E. coli isolates. Even the figure is number 2, but you said Figure 1. | Figure 1 is the electrophoresis image  Figure 2 is the chart  It now says Figure 2: Detection of ESBL in E. coli isolates. |
