# Supplementary material for: Extended spectrum β-lactamase (ESβL)-producing E. coli causing urinary tract infection among pregnant women and pediatric patients in public hospitals in northern Jordan
Source: PLoS One. 2025 Mar 31;20(3):e0320292. doi: 10.1371/journal.pone.0320292 (PMC11957286; doi:10.1371/journal.pone.0320292)
Supplement: Attachment — Submitted filename: Reviewer comment for PLOS ONE.docx [file pone.0320292.s003.docx]

Reviewer comment

Although the paper focuses on an important public heath problem and used advanced methods for gene detection it has major problems as follows:

**Generally in the material and method section**

The paper does not put the study area; study design and study period; source population; study population; inclusion and exclusion criteria of participants clearly.

The sample collection, bacterial identification and antibiotic susceptibility test are not described in detail and clear manner.

The reference guideline (CLSI or…) used for to say resistance, intermediate and sensitive is not mentioned.

The pcr condition, cycles, volumes …… totally not described.

The data analysis, quality control not included

**In result section**

The results written for each section is too shallow please elaborate more.

The tables for AST result (4 and 5) only described in percentage please include the number or frequency

The table title for table 4 it should be above the table

All the table titles and figure legends are too shallow. The tables and figures should be self explanatory.

No conclusion after the discussion

In general the manuscript has many problems which are difficult to comment line by line.
