# Supplementary material for: Extended spectrum β-lactamase (ESβL)-producing E. coli causing urinary tract infection among pregnant women and pediatric patients in public hospitals in northern Jordan
Source: PLoS One. 2025 Mar 31;20(3):e0320292. doi: 10.1371/journal.pone.0320292 (PMC11957286; doi:10.1371/journal.pone.0320292)
Supplement: Attachment — Submitted filename: Reviewer comment aftrer revision.docx [file pone.0320292.s005.docx]

Reviewer comment

The authors have addressed most of the previous reviewer comments; however, the manuscript still has many problems.

Line 1: insert symbol for beta before word lactamase

Line 23-24: remove this sentence “and is responsible for 60-80% of these infections.”

Line 24-25: replace this sentence “UTI is classified based on clinical presentation and diagnosis into upper UTI and lower UTI” with this: “based on the clinical presentation and diagnosis UTI classified into upper and lower UTI”

Line 32,33,39 ,126,127,133 and other parts of the manuscript write the scientific name of bacterial isolates correctly: species name is not started in capital letter: write *Klebsiella pneumoniae* correctly

"Line 40 states, 'Multiple antibiotic resistance.' What does it mean? Does it refer to 'multidrug resistant'?"

Line 41: “0.62” change it in to percentage

Line 37-44: The conclusion section in the abstract appears to present specific results rather than a conclusion. Please provide a general conclusion of the study based on your results.

I started assessing the manuscript line by line, but I couldn’t reach the end because it is full of errors

General comments

Some scientific name of bacterial isolates note written correctly.

There no definition or criteria for multidrug resistance isolates

Conclusion statement in the abstract and the conclusion section are completely different.

In the method section

The ethical statement is included in the study design section

Please list the names of the antibiotics used in a statement rather than in a table.

There no reference putted for the for CLSI in the method section

Please provide the detailed PCR conditions in a statement rather than in a table, starting from the initial denaturation to the final extension.

The author comparing the prevalence of bacterial isolates and antibiotic resistance results among different groups without including statistical significance values

In some cases, the author uses only the genus, while in others, species of isolates are used. See Tables 4, 5, 6, and others.

Finally, although this study used advanced methods like VITAKE and PCR, the authors need a well-experienced microbiologist to understand the results and rewrite the manuscript. Moreover, the author needs individuals for proofreading in English for grammar, spelling, coherence, and flow of sentence.
