# Supplementary material for: Extended spectrum β-lactamase (ESβL)-producing E. coli causing urinary tract infection among pregnant women and pediatric patients in public hospitals in northern Jordan
Source: PLoS One. 2025 Mar 31;20(3):e0320292. doi: 10.1371/journal.pone.0320292 (PMC11957286; doi:10.1371/journal.pone.0320292)
Supplement: Attachment — Submitted filename: Response to Reviewer Comments.docx [file pone.0320292.s009.docx]

| Comment | Reply |
| --- | --- |
| Regarding reviewing the revision by a microbiologist | One of the main authors who is a known microbiologist has reviewed and approved the manuscripts and helped in re writing it, with thanks |
| The gel picture is snipped one put picture that show the ends of the gel | All figures were updated, Figures 1 to 5. |
| Figure2 is not labeled like ladder (label as 100bp, 200bp, 300bp… up to the target gene) | All figures were updated, Figures 1 to 5. |
| Genes detected not labeled in the figure (look other publications) | All figures were updated, Figures 1 to 5. |
| Discussion should start by explaining your work not others | Updated |
| Line 185-191 shows the comparison of others work without even indicating your work | Added “ In this study, the occurrence of UTI in children was higher than adults (60% and 40% respectively).” |
| The result of molecular work not well written both in the result and discussion section | Changed |
| Figure 1 only shows result of two genes what about the other two even if they were not detected they should be indicated as zero in frequency or percentage | Percentages were Added |
| Table titles are not included the study area and time. | See methodology section for study area and time |
| The term "multiple antibiotic resistance" is incorrect and should be replaced with "multidrug resistance (MDR)." Please use the provided reference for identifying and counting MDR isolates. | Changed |

Dear Editor;

The editor's comments, and the reviewers’ comments are highly appreciated, and valued. Please find the response point by point and the changes made. We did our best effort to adjust and improve the quality of this article. Your insight and input is much appreciated.

Reviewer 1

Reviewer 2

| Comment | Reply |
| --- | --- |
| Background: The sentence "Based on the clinical presentation and diagnosis, UTI is classified into upper and lower UTI" should be removed. Instead, focus on the main gaps identified that led to the selection of the study title. | This was requested by the first reviewer |
| Methodology: The methodology section is underdeveloped. Please expand it to include the total number of study participants, the sampling technique used, and the methods for data entry and analysis. | updated |
| Results: Acceptable as is. | Thank you |
| Conclusion: The conclusion section is fine, but it is recommended to remove any percentage descriptions. | done |
| The introduction does not provide a clear explanation of extended spectrum β-lactamase (ESβL)-producing E. coli in relation to urinary tract infections (UTIs) among pregnant women and pediatric patients. Revise to make this connection clearer. | See lines 75-78 in the introduction with thanks |
| Clarify how the study title was selected. What specific gaps or issues prompted this title? | See methodology please |
| Provide scientific reasoning or evidence to explain the occurrence of UTI relapse in the study population. | See conclusion please |
| Use updated references to strengthen the introduction. | Checked and done thanks |
| Overall, rewrite the introduction to improve clarity and focus on the study's objectives and significance. | Made changes thanks for the comment |
| The ethical considerations should not be included under "Study Setting and Population". Please move it to a separate section for clarity. | Changed |
| Key sections are missing, including source and study population, sample size determination, sampling technique, and data analysis methods. Include a description of the data collection procedure, along with details on data entry and data analysis. | Sampling technique is mentioned, line 101 “Samples were collected from each patient using the midstream clean-catch technique [8]” |
